# 4-Methoxydalbergione suppresses growth and induces apoptosis in human osteosarcoma cells *in vitro* and *in vivo* xenograft model through down-regulation of the JAK2/STAT3 pathway

**DOI:** 10.18632/oncotarget.6873

**Published:** 2016-01-09

**Authors:** Kyung-Ran Park, Hyung-Mun Yun, Tran-Hong Quang, Hyuncheol Oh, Dong-Sung Lee, Q-Schick Auh, Eun-Cheol Kim

**Affiliations:** ^1^ Department of Oral and Maxillofacial Regeneration, Kyung Hee University, Seoul, Republic of Korea; ^2^ Department of Oral and Maxillofacial Pathology, School of Dentistry and Research Center for Tooth and Periodontal Regeneration (MRC), Kyung Hee University, Seoul, Republic of Korea; ^3^ Institute of Pharmaceutical Research and Development, College of Pharmacy, Wonkwang University, Iksan, Korea; ^4^ Department of Biomedical Chemistry, College of Health and Biomedical Science, Konkuk University, Chung-Ju, Korea; ^5^ Department of Oral Medicine, School of Dentistry, Kyung Hee University, Seoul, Republic of Korea

**Keywords:** Dalbergia odorifera, 4-methoxydalbergione, osteosarcoma, JAK2/STAT3, MAPK

## Abstract

Although the heartwood of Dalbergia odorifera T. Chen (Leguminosae) is an important source of traditional Korean and Chinese medicines, the effects of novel compound methoxydalbergione (4-MD) isolated from *Dalbergia odorifera* was not reported. Herein, we investigated the effects of the 4-MD *in vitro* and *in vivo* against osteosarcoma cells and its molecular mechanisms. 4-MD inhibited the proliferation of osteosarcoma cells and induced apoptosis as evidenced by Annexin V ^+^ and TUNEL ^+^ cells. This apoptosis was accompanied by upregulation of apoptotic proteins (procaspase-3 and PARP), but downregulation of anti-apoptotic proteins (Bcl-2, Bcl-xL, and Survivin). 4-MD inhibited phosphorylation of JAK2 and STAT3 with the inactivation of mitogen-activated protein kinases (MAPKs) and CREB, and the upregulation of PTEN in osteosarcoma cells. Importantly, 4-MD reduced colony formation in soft agar and inhibited tumor growth in mice xenograft model in association with the reduced expression of PCNA, Ki67, p-STAT3, and Survivin. Taken together, the present study for the first time demonstrates that 4-MD exerts *in vitro* and *in vivo* anti-proliferative effects against osteosarcoma cells through the inhibition of the JAK2/STAT3 pathway, and suggest the potential for therapeutic application of 4-MD in the treatment of osteosarcoma.

## INTRODUCTION

Osteosarcoma is the most common bone malignancy, accounting for about 60% of malignant bone tumors diagnosed in the children and adolescents with an aggressive local pattern of growth and high metastatic potential [1-2]. Although the combination of surgery and chemotherapy has improved osteosarcoma treatment dramatically, no substantial change in survival has been seen over the past 20 years [3]. Therefore, novel therapeutic strategies for osteosarcoma are required.

Apoptosis is a form of cell death coordinated by a network of genes and is a key target in the development of new anti-cancer therapies [4-5]. Intracellular activation of caspases leads to the degradation of cellular proteins to maintain cell survival and death, and also apoptosis is regulated by the proapoptotic B-cell lymphoma protein-2 (Bcl2) family of proteins, such as Bax, Bid, and Bak, and by the anti-apoptotic Bcl2 family of proteins such as Bcl2 and Bcl-xL, and Survivin [6]. The signal transducers and activators of transcription 3 (STAT3) is a latent transcription factor that resides in the cytoplasm [7]. STAT3 phosphorylation is mediated through the activation of non-receptor protein tyrosine kinases family of Janus-like kinase (JAK). The JAK2/STAT3 pathway has shown to have roles in the oncogenesis of several cell types [8-9]. JAK2/STAT3 signaling has been shown to regulate the expression of genes that participate in oncogenesis such as apoptosis inhibitors (Bcl-xl, Bcl-2, and Survivin) and cell cycle regulators [10]. Thus, it is suggested that targeting JAK2/STAT3 proteins may represent an important therapeutic target for novel cancer therapy.

*Dalbergia odorifera* (*D. odorifera*) is mainly distributed in China and, its heartwood is used as a traditional medicine in China and Korea for treating blood disorders, ischemia, swelling, necrosis, and rheumatic pain [11]. Previous studies have reported that *D. odorifera* possesses a variety of beneficial properties including antioxidant, antimicrobial, antiinflammatory, and antitumor activities in diverse cells types [12-17]. We previously demonstrated that flavonoids extracted from *D. odorifera*, exhibited cytoptotective and anti-inflammatory properties [17-18]. Recently, we reported that 2,4,5-trimethoxyldalbergiquinol (TMDQ) isolated from *D. odorifera* promotes osteoblastic differentiation [19].

Although we isolated and identified 4-methoxydalbergione (4-MD) from the heartwood of *D. odorifera*, its pharmacological effects in osteosarcoma have not been reported yet. This study investigated whether 4-MD can mediate its anti-proliferative and apoptotic effects in human osteosarcoma cells through the suppression of the JAK2/STAT3 pathway. In addition, the effects of the 4-MD *in vivo* xenograft models of osteosarcoma were assessed.

## RESULTS

### 4-MD inhibits cell growth in osteosarcoma cells

**Figure 1:**
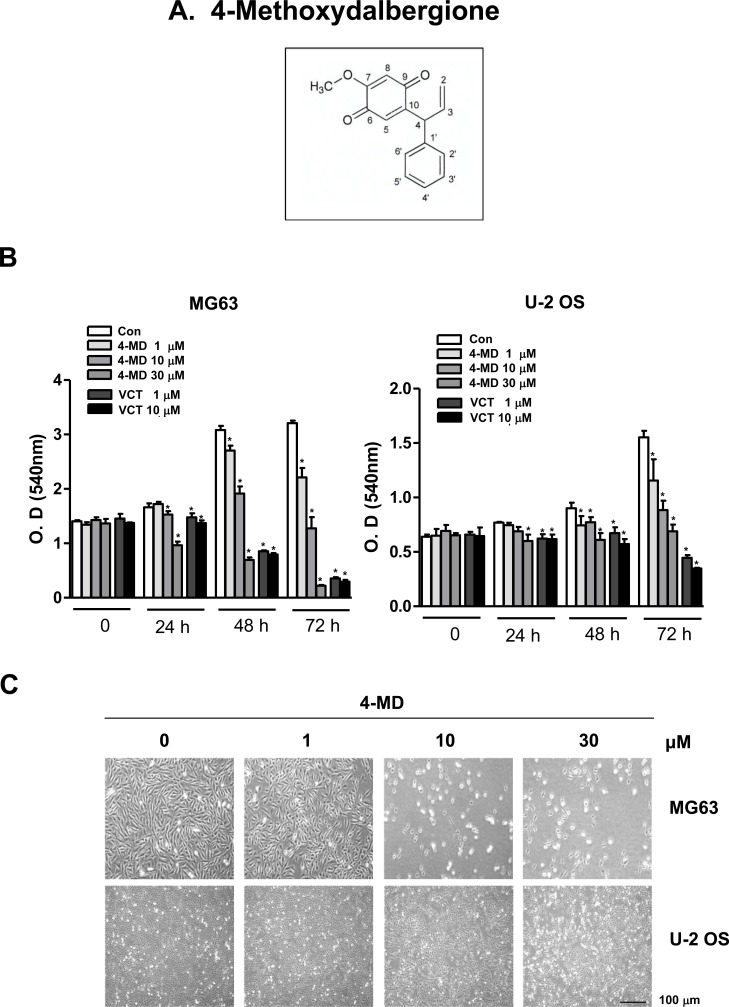
Effects of 4-MD on cell growth in osteosarcoma cells **A.** Chemical structures of 4-Methoxydalbergione (4-MD). **B.** After MG63 and U-2 OS (5 × 10^3^ cells/well) were seeded onto 96-well plates, the cells were cultured in the indicated concentration of 4-MD or Vincristine (VCT) for 72 h. Cell proliferation of MG63 and U-2 OS was measured *via* MTT assay. **C.** After treatment with 4-MD for 24 h, the morphological changes of MG63 and U-2 OS were observed. The results are representative of three independent experiments performed. *: statistically significant difference compared to the control group (*p* < 0.05).

4-MD was isolated from dried heartwoods of *Dalbergia odorifera* (Figure 1A). In order to investigate the inhibitory effect of 4-MD on cell growth of osteosarcoma MG63 and U-2 OS cells, the cells were treated with 1, 10, and 30 μM concentrations of 4-MD for 24 h, 48 h and 72 h, and then analyzed using a MTT assay. The inhibitory effects of 4-MD were compared between aggressively growing osteosarcoma (MG63) and mildly growing osteosarcoma (U-2-OS) cells. As shown Figure 1B, cell growth inhibitory effects were significantly exhibited in concentration-dependent manners both in MG63 and U-2-OS cells. However, 4-MD effectively inhibited cell growth in MG63 cells compared to U-2-OS cells (Figure 1B). Compared to Vincristine (VCT), a commercial chemotherapeutic agent, the inhibitory effects of 4-MD quickly appeared at 24 h, as well as 4-MD more effectively suppressed at 48 h and 72 h than VCT (Figure 1B). Morphologic observation clearly showed that the MG63 cells were gradually reduced in size and changed into a small round single cell shape by the treatment of 4-MD in a dose dependent manner compared to U-2-OS cells (Figure 1C), and thus MG63 cells were chosen in all subsequent experiments.

### 4-MD induces early and late apoptosis

**Figure 2:**
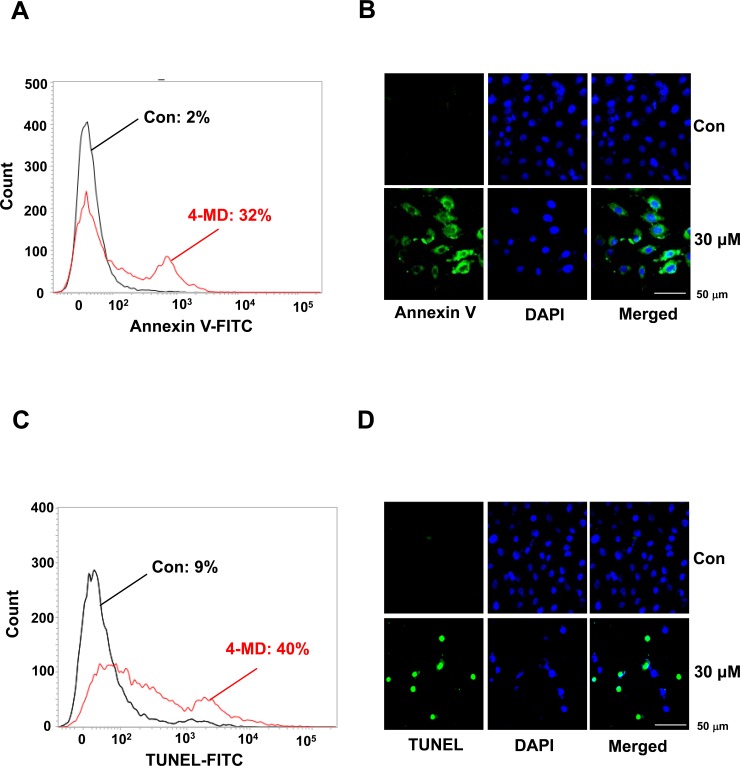
Effects of 4-MD on apoptotic cell death in MG63 cells **A.**, **B.** The cells were treated with 4-MD for 24 h, then incubated with a FITC-conjugated annexin V antibody and analyzed by a flow cytometry (A) and under a confocal microscope (B). **C.**, **D.** 24 h after treatment with 4-MD, the cells were fixed, incubated using TUNEL reaction solution, and then analyzed by flow cytometry (C) and under a confocal microscope (D). These data were representative of three independent experiments.

To determine whether the 4-MD-induced growth inhibition of ostesarcoma cells was associated with the induction of apoptosis, cells were treated with 4-MD and assessed using two apoptosis assays, Annexin V-FITC and TUNEL assays. As shown in Figure 2A, the percentage of apoptotic cells by annexin V-FITC assay was increased in 4-MD-treated osteosarcoma cells as compared to control in a dose dependent manner (Supplementary Figure 1A). A characteristic of apoptosis was present in 4-MD treated cells by fluorescent microscopy after annexin V staining (Figure 2B). When TUNEL assays were performed to assess DNA fragmentation as a late event in the process of apoptosis in osteosarcoma cells after treatment with 4-MD, the dose-dependent TUNEL-positive cells were increased (Figure 2C, 2D and Supplementary Figure 1B).

### 4-MD regulates apoptotic regulatory proteins

**Figure 3:**
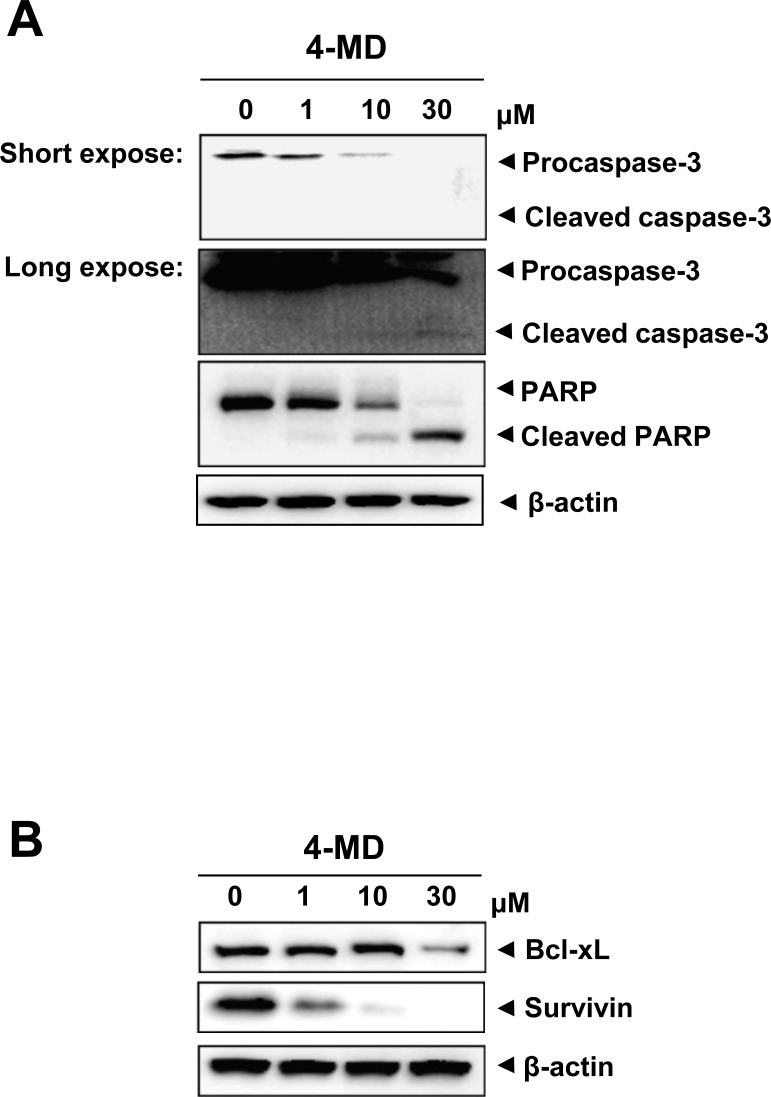
Effects of 4-MD on expression of apoptotic regulatory proteins in MG63 cells **A.**, **B.** The cells were treated with the indicated concentration of 4-MD for 24 h, and then equal amounts of lysates were analyzed by Western blotting using antibodies against caspase-3 and PARP (A), or Bcl-2 and Survivin (B). β-actin was used as a loading control. These data were representative of three independent experiments.

To investigate the underlying mechanisms involved in 4-MD-induced apoptosis, the change in the expression levels with various apoptotic and antiapoptotic proteins was analyzed. 4-MD induced the cleavage of procaspase-3 and PARP as seen by the disappearance of the procaspase-2 and PARP band, and appearance of its cleavage products (Figure 3A). In contrast, 4-MD suppressed the expression of antiapoptotic proteins such as Bcl-xL and Survivin in a concentration-dependent manner (Figure 3B).

### 4-MD inhibits the JAK2/STAT3 pathways *via* the inactivation of MAPKs and the upregulation of PTEN

**Figure 4:**
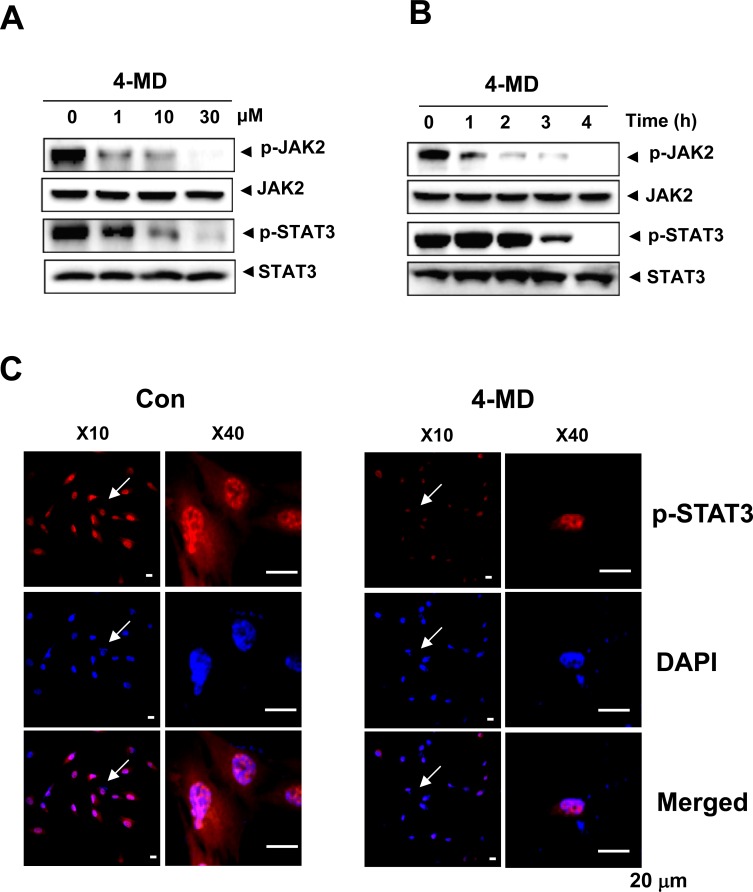
Effects of 4-MD on the JAK2/STAT3 pathway **A.**, **B.** The cells were treated with the indicated concentration (1, 10, and 30 μM) of 4-MD for 4 h (A) or treated with the indicated time (1, 2, 3, and 4 h) of 30 μM 4-MD (B). The equal amounts of lysates were analyzed by Western blotting and detected with antibodies against phospho-JAK2 (p-JAK2), JAK2, phospho-STAT3 (p-STAT3), and STAT3. **C.** 4 h after 4-MD treatment, the cells were fixed and permeabilized. p-STAT3 (*red*) was immunostained with rabbit anti-p-STAT3 antibody, followed by Alex568-conjugated secondary antibody. And then the cells were stained with DAPI (a nuclear marker, *blue*). The *bottom panels* show the merged images of the *first* and *second panels*. These data were representative of three independent experiments.

To evaluate the effects of 4-MD on activation of STAT3 and its upstream JAK2 pathway in osteosarcoma cells, Western blot analysis was performed. As shown in Figure 4A, 4-MD inhibited the constitutive phosphorylation of JAK2 and STAT3 in a dose-dependent manner, with maximum inhibition occurring at 30 μM. Time course studies also indicate that 30 μM 4-MD dramatically blocked phosphorylation of JAK2 and STAT3 with maximum inhibition occurring at 4 h (Figure 4B). Because nuclear translocation is central to the function of transcription factors and it is not certain whether phosphorylation is mandatory for nuclear transport of STAT3 and its oncogenic functions, it was determined whether 4-MD can suppress nuclear translocation of STAT3. 4-MD inhibited the translocation of STAT3 to the nucleus in osteosarcoma cells (Figure 4C)

**Figure 5:**
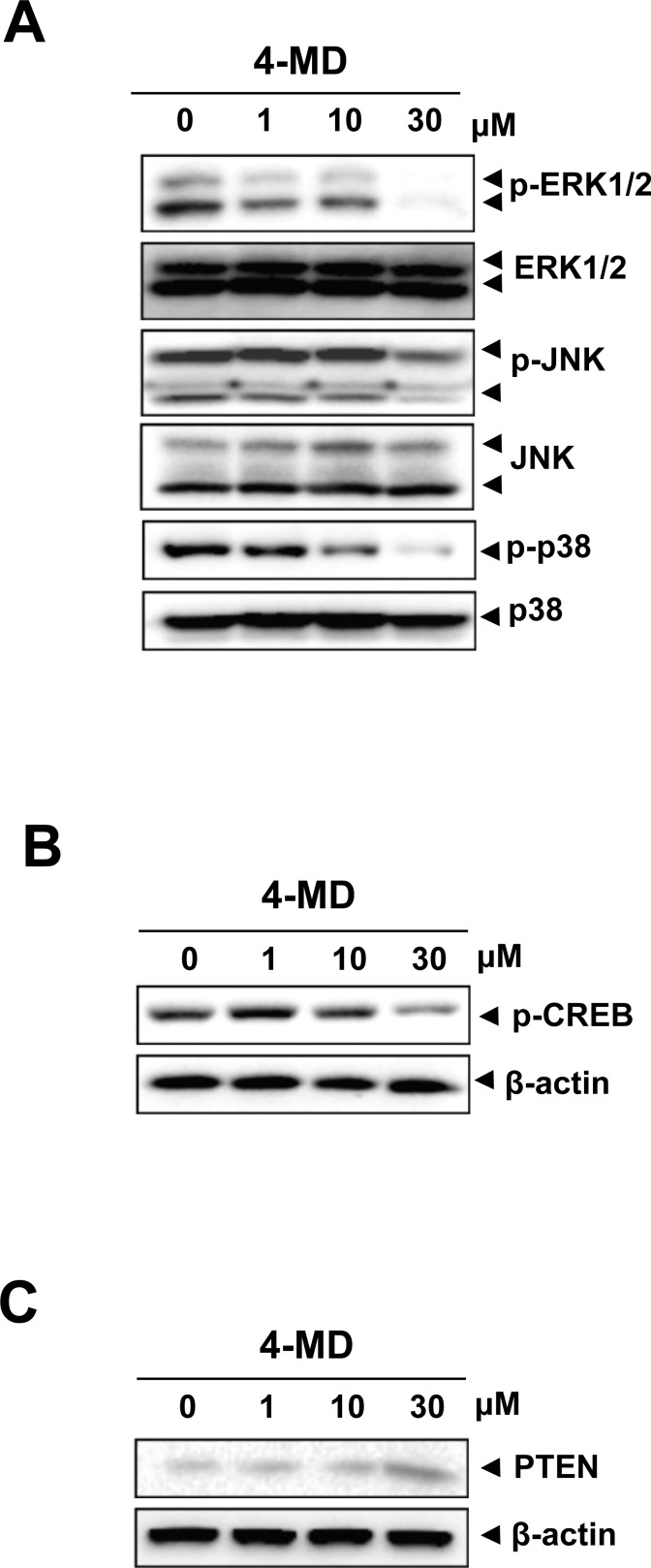
Effects of 4-MD on the MAPKs pathway and PTEN expression **A.**-**C.** The cells were treated with the indicated concentration of 4-MD for 30 min (A) or 24 h (B, C), and then equal amounts of lysates were detected with antibodies against phospho-ERK (p-ERK), ERK, phospho-JNK (p-JNK), phospho-p38 (p-p38), and p38 (A), or phospho-CREB (p-CREB) and CREB (B), or PTEN and β-actin (C). These data were representative of three independent experiments.

Next, the effects of 4-MD on the activation of mitogen-activated protein kinase (MAPK), and cAMP response element-binding protein (CREB) were investigated. Western blot analysis showed that treatment of the cells with 4-MD significantly reduced the activation of ERK1/2, JNK and p38 MAPK as well as CREB in a dose dependent manner (Figure 5A, 5B). In order to determine the downstream consequences of MAPK and CREB, the ability of 4-MD on expression of PTEN (phosphatase and tensin homolog deleted on chromosome ten) was examined. The results showed a concentration-dependent increase of PTEN in osteosarcoma cells treated with 4-MD, as compared with that in untreated cells (Figure 5C).

### 4-MD inhibits *in vitro* colony formation and *in vivo* tumor size in xenograft nude mice

**Figure 6:**
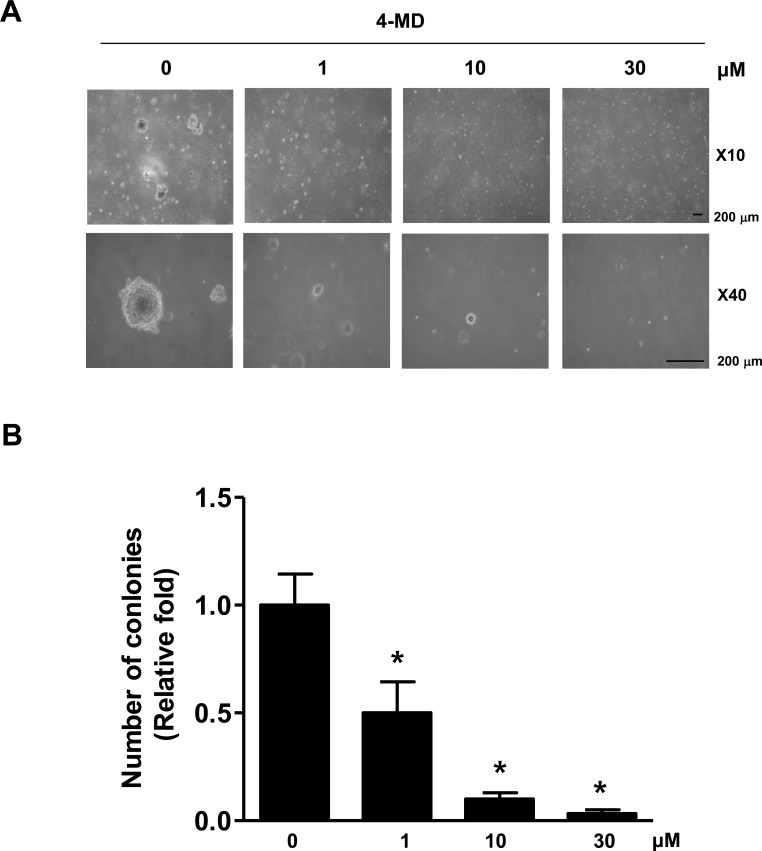
Effects of 4-MD on colony formation in MG63 cells **A.**, **B.** The cells (1.5 × 10^4^ cells/well) were plated in the top layer and incubated with the indicated concentration of 4-MD for 14 days. Colony formation was observed under a light microscope (A) and the colonies were counted. The bar graph was normalized to control (B). These data are representative of three independent experiments. *: statistically significant difference compared to control (*p* < 0.05).

We next investigated the effect of 4-MD on anchorage-independent growth by soft agar colony formation, which is a good model to study tumorigenicity and is closely associated with the transformed property of cells [20]. As shown in Figure 6, exposure of 4-MD caused concentration-dependently significant reduction in anchorage-independent growth and colony formation compared with vehicle treated controls.

**Figure 7:**
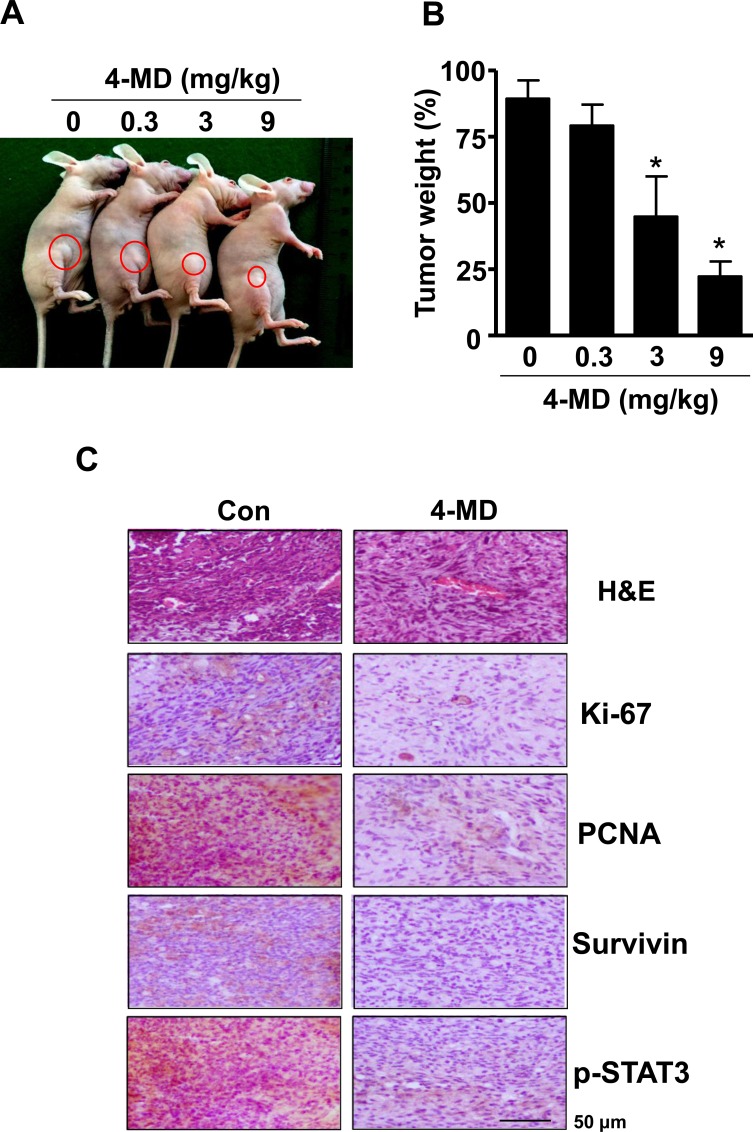
Effect of 4-MD on tumor growth in xenograft model **A.**, **B.** MG63 cells were injected into the right lower flanks in BALB/c athymic nude mice. All data indicate tumors formed on the right lower flanks. Images of MG63 bearing mice (A) and tumor weight (B). **C.** Tumor sections of control and 4-MD (3mg/kg) treated MG63 bearing mice were analyzed by H&E stain and expression of proteins by immunohistochemistry. The resultant tissues were developed with DAB, and counterstained with hematoxylin. Scale bar indicates 50 μm.

On the basis of our *in vitro* studies, we finally examined the *in vivo* antitumor activities of 4-MD in a xenograft mouse models. Representative tumors in the xenograft mice treated with or without 4-MD are shown in Figure 7A. Moreover, 4-MD significantly decreased by 22.25 ± 11.46 % of the tumor weight compared to control (Figure 7B). To determine whether growth inhibition and apoptosis is responsible for the observed antitumor activity of 4-MD, immunohistochemistry for proliferation, STAT3 signaling, and antiapoptotic proteins were examined in xenograft tissue. Results showed that 4-MD effectively suppressed the expression of proliferation marker (Ki-67 and Proliferating cell nuclear antigen (PCNA), ant-apoptotic molecule (Survivin), and therapeutic target molecule (p-STAT3) in tumor tissues (Figure 7C).

## DISCUSSION

Despite recent therapeutic advances, osteosarcoma remains one of the most lethal and treatment resistant solid tumors. As a part of our ongoing effort for the identification of natural products with anti-cancer effects *in vitro and in vivo*, we present here first time that 4-MD inhibits the growth and induces apoptosis of osteosarcoma cells through the suppression of the JAK2/STAT3 pathway, and inhibits growth of osteosarcoma cells in a xenograft mouse model.

To date, no data have been reported regarding the effects of 4-MD in osteosarcoma cells. To gain insight into the effects of 4-MD, we evaluated the effects of 4-MD in two commonly used osteosarcoma cell lines, U-2 OS and MG 63. Our cell proliferation assays and two apoptosis assays showed that 4-MD strongly inhibited the proliferation and induced apoptosis of osteosarcoma cells in a dose-dependent manner. The results suggested that the death of osteosarcoma cells treated with 4-MD was a consequence of cell apoptosis caused by 4-MD. These results was supported by 4-MD treatment upregulation of apoptotic proteins (procaspase-3 and PARP), but downregulation of anti-apoptotic proteins (Bcl-2, Bcl-xL, and Survivin).

JAK2/ STAT3 signaling regulates several important pathways in tumorigenesis including cell cycle progression, apoptosis, tumor invasion, and metastasis [21-22]. Thus, activated JAK2/STAT3 signaling has been extensively validated as a new molecular target for the treatment of human solid tumor [10]. For example, natural products inhibit JAK2/STAT3 signaling and induce apoptosis in various cancer cells [10, 23]. In the present study, 4-MD treatment directly inhibited the phosphorylation of JAK2 and the downstream phosphorylation of STAT3. Moreover, 4-MD also down-regulated the expression of the STAT3 target proteins, Bcl-xL, and Survivin. These results indicate that JAK2/STAT3 signaling is involved in the anti-osteosarcoma effect of 4-MD.

MAPK signaling cascades is also important for activation of STAT3 [24-25]. CREB is a transcription factor which is a common critical MAPK downstream pathways [26]. PTEN is a major negative regulator of the PI3-K/AKT signal pathway involved in tumorigenesis [27]. PTEN can also regulate JAK2/STAT3 signaling, which can alter its ability to regulate the transcription of genes [27-28]. Our results showed that 4-MD reduced the phosphorylation levels of ERK, JNK, and p38 as well as CREB, and induced significant increase in PTEN expression. These results suggested that 4-MD can regulate the activity of CREB and MAPK, and consequently increases the expression of PTEN, which leads to STAT3 inactivation and osteosarcoma cells growth.

In the present study, 4-MD reduces anchorage-independent growth of ostesarcoma cells, and inhibits tumor growth in a xenograft nude mouse model. Ki-67 protein is expressed in proliferating cells during all active phases of the cell cycle and is regarded as the most promising biomarker for cell proliferation [29]. PCNA is involved in eukaryotic DNA synthesis and plays an important role in the cell cycle regulation [30]. Expression of PCNA is significantly increased in the process of malignant transformation of normal epithelium and is considered as a cell proliferation marker [30-31]. Concomitant with the results obtained for *in vivo* tumor growth inhibition, we also observed decreased proliferation as documented by PCNA and Ki-67 immunostaining, and increased apoptosis as documented by decreased Survivin expression within tumor tissues. Furthermore, immunohistochemical analysis confirmed the downregulation of p-STAT3 following treatment with 4-MD in *vivo*, which was consistent with our findings *in vitro*. Thus, our results suggest that 4-MD inhibits the JAK2/STAT3 signaling, which in turn leads to suppression of tumor growth *in vitro and in vivo*.

In conclusion, the present study is the first report that 4-MD shows profound anti-cancer activity *in vitro* and *in vivo, and also* induces cell apoptosis in osteosarcoma cells by inhibiting the *JAKT2/STAT3* pathway. These findings suggest that 4-MD can be potentially applicable for therapeutic effects of osteosacoma.

## MATERIALS AND METHODS

### Isolation and identification of 4-Methoxydalbergione

As previous reported [32], Dried heartwoods of *Dalbergia odorifera* (1.65 kg) were extracted with EtOH by untrasonics for 1 h. After concentrated *in vacuo*, the EtOH extract (200 g) was suspended in H_2_O and partitioned with EtOAc to give EtOAc (DOE, 180 g) and aqueous fractions. Fraction DOE was subjected to a silica gel column chromatography (CC) and eluted with acetone in *n*-hexane (5-50%, step-wise) and washing with MeOH to yield seven subfractions (DOE1-6). Subfraction DOE3 was separated by a silica gel CC, eluting with *n*-hexane-CH_2_Cl_2_ (1:2) to give five subfractions (DOE31-5). Subfraction DOE32 was further separated by a silica gel CC, eluting with *n*-hexane-EtOAc (5:1) to give 4-Methoxydalbergione (8 mg). The structure of this compound was elucidated by comparative analysis of the ^1^H- and ^13^C-NMR spectroscopic data with those reported in the literature [32].

**4-Methoxydalbergione**: yellow powder. ^1^H-NMR (CDCl_3_, 400MHz) δ 5.00 (1H, dt, *J* = 1.2, 16.8 Hz, H-2a), 5.26 (1H, dt, *J* = 1.2, 10.4 Hz, H-2b), 6.08 (1H, ddd, *J* = 6.8, 10.4, 16.8 Hz, H-3), 4.92 (1H, br d, *J* = 6.8 Hz, H-4), 6.47 (1H, d, *J* = 1.2 Hz, H-5), 5.89 (1H, s, H-8), 7.29 (2H, d, *J* = 8.4 Hz, H-2′ and H-6′), 7.18 (2H, t, *J* = 8.4 Hz, H-3′ and H-5′), 7.23 (1H, t, *J* = 8.4 Hz, H-4′), 3.77 (3H, s, 7-OCH_3_). ^13^C-NMR (CDCl_3_, 100MHz) δ 118.1 (C-2), 137.1 (C-3), 46.9 (C-4), 131.4 (C-5), 182.2 (C-6), 158.3 (C-7), 107.7 (C-8), 186.1 (C-9), 150.9 (C-10), 139.2 (C-1′), 128.6 (C-2′ and C-6′), 128.4 (C-3′ and C-5′), 127.0 (C-4′), 56.1 (7-OCH_3_).

### Cell culture

MG63 and U-2 OS cells were grown in Dulbecco's modified Eagle medium (DMEM) supplemented with 10% fetal bovine serum (FBS), penicillin (100 units/ml), and streptomycin (100 μg/mL) at 37°C in a humidified atmosphere of 5% CO_2_ and 95% air. Direct seeding of cells (5 × 10^5^ cells/ 6 well plate) was performed For the experiments. After overnight incubation, culture medium was replaced with fresh DMEM and cells were treated with various concentration of 4-MD.

### Cell proliferation

Cell proliferation was measured by an 3-[4,5-dimethylthiazol-2-yl]-2,5-diphenyltetrazolium bromide (MTT) assay to detect NADH-dependent dehydrogenase activity as previously described [33]. Fifty microliters of MTT solution (5 mg/mL) in 1X phosphate-buffered saline (PBS) was directly added to the cells, which was then incubated for 2 h to allow MTT to metabolize to formazan. Absorbance was measured at a wavelength of 540 nm using an enzyme linked immunosorbent assay (ELISA) reader (Beckman Coulter, Fullerton, CA).

### Annexin V analysis

The cells were treated with 4-MD for 24 h and stained by Annexin V conjugated to FITC. The cells were washed and observed with a flow cytometry (BD FACSVerse, BD Biosciences, San Jose, CA). For the confocal microscope assay, the cells were seeded onto poly-L-lysine-coated slides, stained by Annexin V conjugated to FITC, and fixed. The cells were then stained with 1 μg/mL DAPI (Sigma-Aldrich, St. Louis, MO) solution for 5 min, washed three times, mounted on glass slides, and viewed on confocal microscopy (Cell Voyager, Yokohama, Japan).

### TUNEL assay

DNA fragmentation was examined by terminal deoxynucleotidyl transferase-mediated FITC-dUDP nick-end labeling (TUNEL). TUNEL assays were performed using the in situ Cell Death Detection Kit (Roche Diagnostics GmbH, Mannheim, Germany) according to the manufacturer's instructions. The cells were seeded onto 6-well plates and treated with 4-MD for 24 h. The cells were collected and washed with 1x PBS. Cell pellets were fixed with 4% paraformaldehyde for 30 min and were permeabilized by 0.2% Triton X-100 for 15 min. After then, the cells were incubated with TUNEL reaction solution for 1 h at 37°C in the dark. The cells were washed and observed with a flow cytometry (BD FACSVerse). For the confocal microscope assay, the cells were seeded onto poly-L-lysine-coated slides, fixed, permeabilized, and added to the TUNEL reaction solution for 1 h at 37°C in the dark. The cells were then stained with 1 μg/mL DAPI (Sigma-Aldrich) solution for 5 min, washed three times, mounted on glass slides, and viewed on confocal microscopy (Cell Voyager).

### Information for antibodies

All sources of antibodies with name of antibodies, dilution ratio of antibodies, catalog numbers, and company in Western blot analysis study as follows: p-ERK1/2 (1:2000, #9101S, Cell Signaling Technology, Beverly, MA), ERK1/2 (1:2000, #9102S, Cell Signaling), p-p38 (1:1000, #9211S, Cell Signaling), p38 (1:1000, #9212S, Cell Signaling), p-JNK (1:500, #9251, Cell Signaling), JNK (1:1000, #9252S, Cell Signaling), JAK2 (1:1000, #3230, Cell Signaling), p-JAK2 (1:1000, #4406, Cell Signaling), STAT3 (1:1000, #12640, Cell Signaling), p-CREB (1:1000, #9191, Cell Signaling), PTEN (1:1000, #9552, Cell Signaling), Survivin (1:1000, #2808, Cell Signaling), Caspase-3 (1:1000, #9665, Cell Signaling), PARP (1:1000, #9542, Cell Signaling), Bcl-xL (1:1000, #sc-7195, Santa Cruz), β-actin (C4) (1:1000, #sc-47778, Santa Cruz), p-STAT3 (1:1000, #sc-8001, Santa Cruz).

For immmo(cyto)histochemistry study: Ki-67 (1:200, #sc-15402, Santa Cruz), PCNA (1:200, #sc-56, Santa Cruz), Survivin (1:200, #2808, Cell Signaling), Bcl-xL (1:200, #sc-7195, Santa Cruz), p-STAT3 (1:200, #sc-8001, Santa Cruz)

### Western blot analysis

Western blot analysis was performed as previously described [34]. Briefly, Cells were washed twice with ice-cold PBS, and lysed in 20 mM Tris-HCl buffer (pH 7.4) containing a protease inhibitor mixture (0.1 mM PMSF, 5 mg/mL aprotinin, 5 mg/mL pepstatin A, and 1 mg/mL chymostatin). Protein concentration was determined by Bradford reagent (Bio-Rad, Hercules, CA). Equal amounts of lysates (20 μg) were resolved on sodium dodecyl-polyacrylamide gel electrophoresis (SDS-PAGE) were transferred to a polyvinylidene fluoride (PVDF) membrane (Millipore, Bedford, MA), and the membrane was blocked with 1X TBS containing 0.05% Tween 20 and 5% skim milk or 2% BSA for 1 h at room temperature. After blocking, the membranes were incubated overnight at 4°C with the respective primary antibodies. The membranes were washed with 1X PBS and incubated with diluted horseradish peroxidase (HRP)-conjugated secondary antibodies (1:10,000, Jackson ImmunoResearch, West Grove, PA) for 1 h at room temperature. After three washes, the membranes were detected using the enhanced chemiluminescence (ECL) kit (Millipore, Bedford, MA).

### Immunocytochemistry

Cells were grown on glass coverslips and incubated with 4-MD. As previously described [35], cells were fixed in 10% formalin for 15 min at room temperature. After washing three times in 1X PBS, the cells were permeabilized with 0.2% Triton X-100 in 1X PBS for 20 min, washed three times in 1X PBS, and then blocked with 5% BSA in 1X PBS for 1 h at room temperature. After then, the cells were incubated with anti-p-STAT3 (1:200, Cell signaling) antibody for overnight at room temperature, washed three times, and incubated with Alexa-568 conjugated secondary antibodies (1:500, Invitrogen, Carlsbad, CA) for 2 h at room temperature. The cells was stained with DAPI (Sigma-Aldrich) and washed three times, mounted on glass slides, and viewed on confocal microscopy (Cell Voyager).

### *In vitro* anti-cancer growth effect in a soft agar assay

2 ml of 0.6% agar was layered in the bottom onto 6-well plates, followed by 2 ml of 0.3% agar as the top layer. MG63 cells were then plated with various concentrations of 4-MD on the top layer. The cells were maintained at 37°C under a 5% CO_2_ atmosphere for 14 days, and the colonies were observed and quantified under a light microscope.

### Ethics statement

All experimental procedures in the current study were approved by Kyung Hee University Animal Care Committee (approval number: KHMC-IACUC 2015-001).

### *In vivo* anti-cancer growth effect in a xenograft animal

Six-week-old male BALB/c athymic nude mice were purchased from Samtako (Osan, Kyoung Gi-Do, Korea). Human osteosarcoma MG63 cells (6 mg/ 1 × 10^6^ cells) were injected subcutaneously (1×10^7^ cells/0.1 mL PBS/animal) into the right-lower flanks of the carrier mice as previously described [36-37]. After 10 days, four groups of mice (*n* = 5) were i.p. injected with 4-MD (0.3, 3, and 9 mg/kg in PBS and 0.01% DMSO) two times a week for 3 weeks. The control group of mice (*n* = 5) were treated with vehicle (PBS and 0.01% DMSO) two times a week for 3 weeks. At the end of the experiment, cervical dislocation was performed for euthanasia.

### Immunohistochemistry

All specimens were fixed in formalin and embedded in paraffin for examination. Sections (5 μm thickness) were stained with hematoxylin and eosin (H&E) and analyzed by immunohistochemistry. The sections were deparaffinized by immersing into xylene solution, rehydrated, subjected to heat-mediated antigen retrieval treatment, washed with distilled water and proceed with immunohistochemical procedure. Endogenous peroxidase activity was quenched by incubation with 1% hydrogen peroxide solution in methanol for 30 min and washed with 1X PBS (Sigma, St. Louis, MO) for 5 min. Next, the sections were blocked with 5% BSA diluted in 1X PBS for 30 min, incubated overnight with specific antibodies at 4°C, and washed 3 times with 1X PBS. The immunological detection was started with incubation in horseradish peroxidase (HRP)-conjugated secondary antibodies (1:500, Jackson ImmunoResearch) for 1 h at room temperature. After washing with 1X PBS, chromogen development was performed with 0.02% 3, 3′-diaminobenzidine tetrahydrochloride (DAB, Vector Laboratories, Burlingame, CA) and slides counterstained with hematoxylin. Finally, sections were dehydrated with ethanol, cleared with xylene, and mounted with Permount (Fisher Scientific, Rockford, IL), and evaluated on a light microscopy (Olympus, Tokyo, Japan).

### Statistical analysis

The data were analyzed using the GraphPad Prism version 5 program (GraphPad Software, Inc., San Diego, CA). Data are presented as mean ± S.E.M. Satistical significance was performed on the data using Newman-Keuls test. A value of *P* < 0.05 was considered to be statistically significant.

## SUPPLEMENTARY MATERIAL FIGURE


