# Supplementary material for: 4-Methoxydalbergione suppresses growth and induces apoptosis in human osteosarcoma cells in vitro and in vivo xenograft model through down-regulation of the JAK2/STAT3 pathway
Source: Oncotarget. 2016 Jan 9;7(6):6960–71. doi: 10.18632/oncotarget.6873 (PMC4872761; doi:10.18632/oncotarget.6873)
Supplement: Supplementary file 1 [file oncotarget-07-6960-s001.pdf]

4-Methoxydalbergione suppresses growth and induces apoptosis in human osteosarcoma cells *in vitro* and *in vivo* xenograft model through down-regulation of the JAK2/STAT3 pathway

Supplementary Material

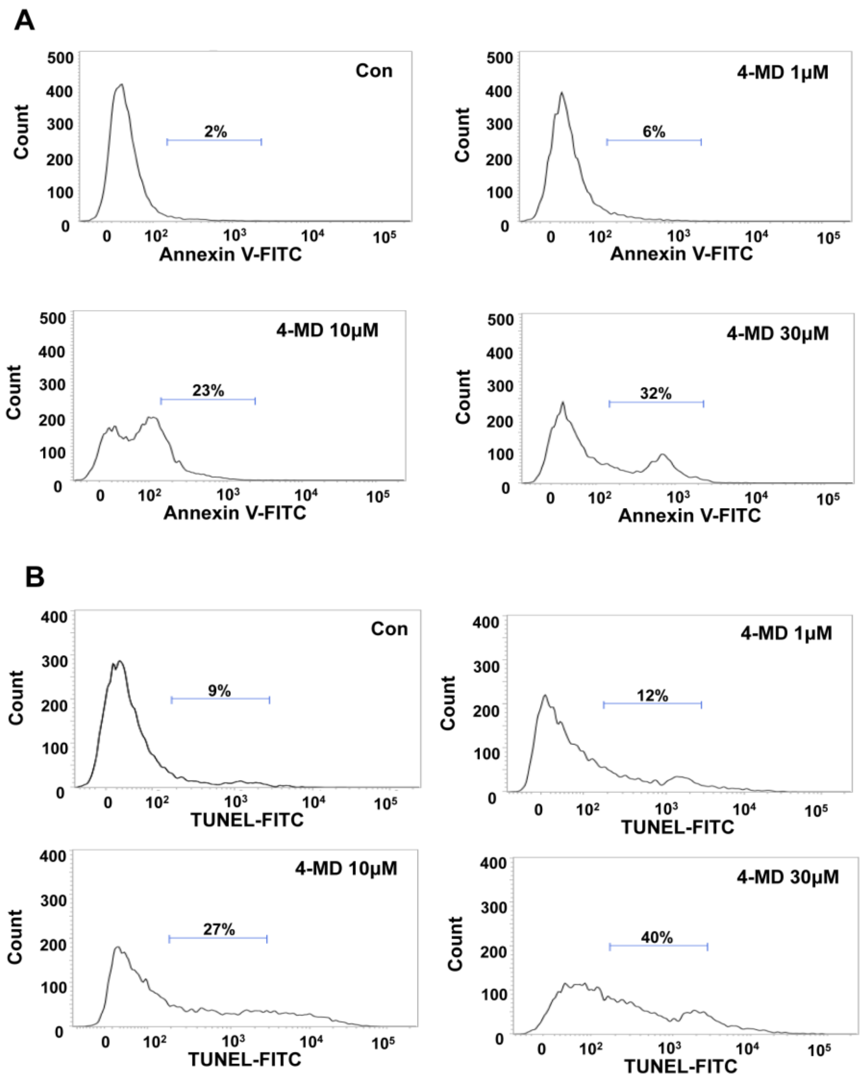

Supplementary Figure 1. Dose dependent effects of 4-MD on apoptotic cell death in MG63 cells. **(A, B)** 24 h after 4-MD treatment (0, 1, 10, and 30 μM), the cells were incubated with a FITC–conjugated annexin V antibody (A) or incubated using TUNEL reaction solution (B), and then analyzed by flow cytometry. These data were representative of three independent experiments.
